# Distributional records of Ross Sea (Antarctica) Tanaidacea from museum samples stored in the collections of the Italian National Antarctic Museum (MNA) and the New Zealand National Institute of Water and Atmospheric Research (NIWA)

**DOI:** 10.3897/zookeys.451.8373

**Published:** 2014-11-03

**Authors:** Paola Piazza, Magdalena Błażewicz-Paszkowycz, Claudio Ghiglione, Maria Chiara Alvaro, Kareen Schnabel, Stefano Schiaparelli

**Affiliations:** 1 Department of Earth, Environmental and Life Sciences (DISTAV), University of Genoa, Genoa, Italy; 2 Italian National Antarctic Museum (MNA, Section of Genoa), University of Genoa, Genoa, Italy; 3 Department of Polar Biology and Oceanobiology, University of Lódź, Banacha, Poland; 4 National Institute of Water and Atmospheric Research (NIWA), Wellington, New Zealand

**Keywords:** Antarctica, Ross Sea, Crustacea, Peracarida, Tanaidacea, MNA, NIWA

## Abstract

Here we present distributional records for Tanaidacea specimens collected during several Antarctic expeditions to the Ross Sea: the Italian PNRA expeditions (“V”, 1989/1990; “XI”, 1995/1996; “XIV”, 1998/1999; “XIX”, 2003/2004; “XXV”, 2009/2010) and the New Zealand historical (New Zealand Oceanographic Institute, NZOI, 1958-1961) and recent (“TAN0402 BIOROSS” voyage, 2004 and “TAN0802 IPY-CAML Oceans Survey 20/20” voyage, 2008) expeditions. Tanaidaceans were obtained from bottom samples collected at depths ranging from 16 to 3543 m by using a variety of sampling gears. On the whole, this contribution reports distributional data for a total of 2953 individuals belonging to 33 genera and 50 species. All vouchers are permanently stored in the Italian National Antarctic Museum collection (MNA), Section of Genoa (Italy) and at the National Institute of Water and Atmospheric Research (NIWA Invertebrate Collection), Wellington (New Zealand).

## Purpose

The aim of the present study is to provide new distributional data for the tanaidaceans collected during past and recent scientific expeditions conducted in the Ross Sea, Antarctica, and now stored at the Italian National Antarctic Museum (MNA), Section of Genoa, Genoa (Italy) or at the National Institute of Water and Atmospheric Research (NIWA Invertebrate Collection), Wellington (New Zealand). The dataset is the second Italian contribution to the Antarctic Biodiversity Information Facility (ANTABIF – http://www.biodiversity.aq) based on materials stored at the Italian National Antarctic Museum (MNA), Section of Genoa, Italy. The first published MNA contribution was by Ghiglione et al. (2013).

## Project details

**Project title:** Ross Sea Tanaidacea in the collections of the Italian National Antarctic Museum (MNA) and National Institute of Water and Atmospheric Research (NIWA)

**Curator and Promoter:** Stefano Schiaparelli

**Personnel:** Magdalena Błażewicz-Paszkowycz, Paola Piazza, Claudio Ghiglione, Maria Chiara Alvaro, Kareen Schnabel, Graham Bird, Stefano Schiaparelli

**Funding:** The tanaidaceans were collected during different Italian and New Zealand research expeditions to the Ross Sea funded by the Italian National Antarctic Research Program (PNRA) and the New Zealand Government, the Ministry for Primary Industries (formerly the Ministry of Fisheries) and the Ocean Survey 20/20 CAML Advisory Group, listed below:

Italian PNRA Project 3.2.1 (Oceanography) (“V” expedition, 1989/1990, R/V “*Malippo*”).

Italian PNRA Project 2a and 2d.2 (Ecology and Biogeochemistry of the Southern Ocean) (“XI” expedition, 1995/1996, R/V “*Italica*”).

Italian PNRA Project 2b.3 (Ecology and Biogeochemistry of the Southern Ocean) (“XIV” expedition, 1998/1999, R/V “*Malippo*”).

Italian PNRA Project Program 2002/8.6 (“The costal ecosystem of Victoria Land coast: distribution and structure along the latitudinal gradient”) (“XIX” expedition, 2003/2004, R/V “*Italica*” 2004).

Italian PNRA Project 2006/08.01 (“The coastal ecosystem of Terra Nova Bay” in the Latitudinal Gradient Program (LGP)) (“XXV” expedition, 2009/2010).

The Ross Sea Endeavour surveys (1958-59 and 1959-60, HMNZS “*Endeavour II*” and 1960–1961, “*Endeavour III*”) conducted by the New Zealand Oceanographic Institute (NZOI, now NIWA) – founded by the New Zealand government.

New Zealand BIOROSS voyage (TAN0402, 2004, R/V “*Tangaroa*”) – funded by NIWA and the New Zealand Ministry of Primary Industries (formerly the Ministry of Fisheries).

New Zealand IPY-CAML voyage (TAN0802, 2008, R/V “*Tangaroa*”) – Census of Antarctic Marine Life programme – funded by the Government of New Zealand and administered by the Ocean Survey 20/20 CAML Advisor Group (Land Information New Zealand and the Ministry of Fisheries, Antarctica New Zealand, Ministry of Foreign Affairs and Trade and NIWA).

**Study area descriptions/descriptor:** The 2953 individuals belonging to 33 genera and 50 species of Tanaidacea were collected in the Ross Sea sector of the Southern Ocean. The bathymetric range was from 16 to 3543 m.

**Design description:** The data was gathered by assembling distributional records for the Ross Sea Tanaidacea species stored at the Italian National Antarctic Museum collection (MNA), Section of Genoa, Genoa (Italy) and at the National Institute of Water and Atmospheric Research (NIWA Invertebrate Collection), Wellington (New Zealand). These samples were obtained in the framework of different past research expeditions, which had different aims and geographical scopes. The earliest records are derived from the NZOI Ross Sea Oceanographic Surveys conducted during three consecutive years between 1958 and 1961 that were part of the New Zealand Antarctic Research Programme with the aim to study the hydrology, geology and biology of the Ross Sea (Bullivant and Dearborn 1967). The main purpose of the “V” (1989/1990), “XI” (1995/1996) and “XIV” (1998/1999) Italian PNRA expeditions was to investigate the distribution and structure of coastal communities in the Terra Nova Bay area. The “XIX” (2003/2004), “XXV” (2009/2010) Italian PNRA expeditions and the New Zealand TAN0402 BIOROSS voyage (2004) aimed at understanding the complex ecosystems along the Victoria Land coast under the Latitudinal Gradient Program framework (LGP, http://www.lgp.aq/). The New Zealand TAN0802 IPY-CAML voyage (2008) aimed at assessing a reference baseline in the Ross Sea, fulfilling the CAML research targets (Schiaparelli et al. 2013).

## Methods

**Figure 1.**
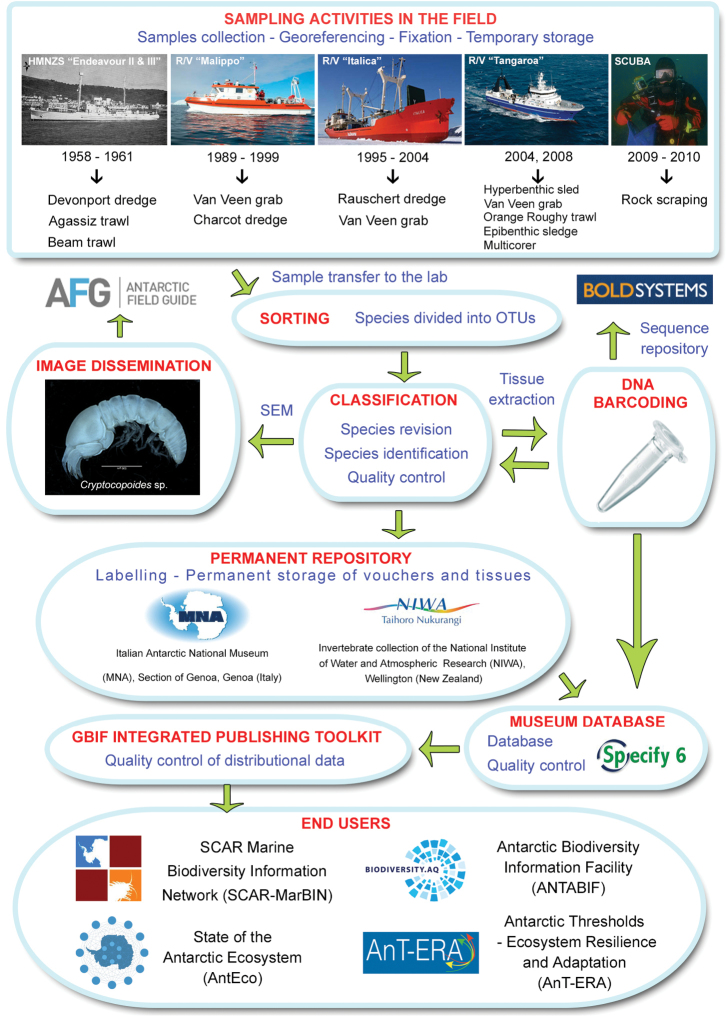
Flowchart depicting major steps in dataset development and publishing.

**Method step description:** See sampling description below and flowchart of Figure 1.

**Figure 2.**
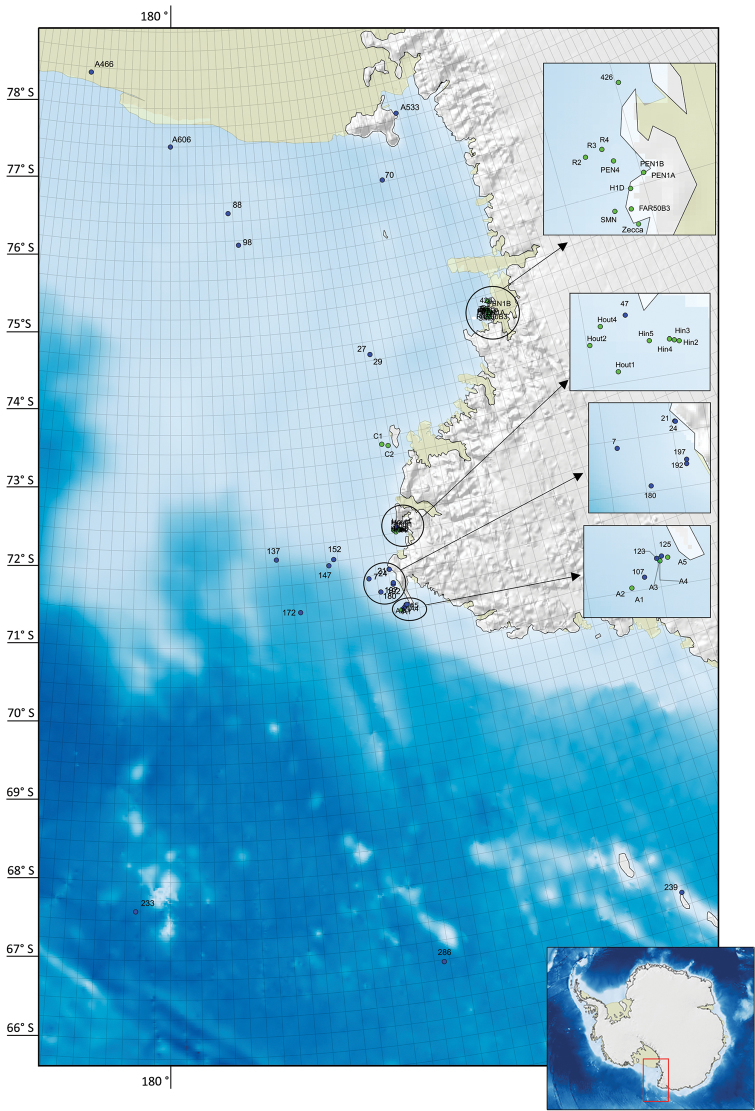
Map of sampling stations. Green dots: samples stored at the MNA. Blue dots: samples stored at the NIWA.

**Study extent description:** The Tanaidacea distributional data considered here originated from 50 sampling stations located in the Ross Sea (comprising its northern archipelagos and seamounts), between 16 and 3543 metres of depth (Fig. 2) and investigated in the framework of different research expeditions from 1958 to 2010. Specifically, these were five PNRA and five New Zealand scientific voyages:

4 species (corresponding to 4 specimens) from three different New Zealand Oceanographic Institute (NZOI) Ross Sea Oceanographic Surveys (stations: A466, A533, A606)

2 species (corresponding to 17 specimens) from the “V” Italian PNRA expedition at Terra Nova Bay (station H1D) on board the R/V “*Malippo*”

2 species (corresponding to 11 specimens) from the “XI” Italian PNRA expedition at Terra Nova Bay (stations 426, PEN 1A, PEN 1B, FAR 50B3) on board the R/V “*Italica*”

1 species (corresponding to 1 specimens) from the “XIV” Italian PNRA expedition at Terra Nova Bay (station PEN 4) on board the R/V “*Malippo*”

40 species (corresponding to 2813 specimens) from the “XIX” Italian PNRA expedition along the Victoria Land Coast (Four different areas: Cape Adare with stations A1, A2, A3, A4, A5; Cape Hallett with stations Hout1, Hout2, Hout4, Hin2, Hin3; Hin4, Hin5; Coulman Island with stations C1, C2; Terra Nova Bay and Cape Russell with stations SMN, R2, R3, R4) on board the R/V “*Italica*”

7 species (corresponding to 80 specimens) from the TAN0402 BIOROSS voyage in the Ross Sea (stations: 7, 21, 24, 47, 107, 123, 125, 180, 192, 197, 239) on board the R/V “*Tangaroa*”

6 species (corresponding to 21 specimens) from the New Zealand TAN0802 IPY-CAML voyage in the Ross Sea (stations: 27, 29, 70, 88, 98, 137, 147, 152, 172, 233, 286) on board of the R/V “*Tangaroa*”

1 species (corresponding to 6 specimens) from the “XXV” Italian PNRA expedition at Terra Nova Bay (stations “zecca”)

**Sampling description:** The material was collected in the framework of several PNRA and NIWA (formerly New Zealand Oceanographic Institute) Antarctic scientific expeditions, through the deployment of a variety of sampling gears. NIWA historical samples (NZOI surveys) were collected by using Devonport dredge, beam trawl and Agassiz trawl. Coastal sampling under the Italian National Antarctic Research Program (PNRA) (“V”, “XI”, “XIV” expeditions) was mainly performed by using Charcot dredge, Van Veen grabs of different sampling volume. Off-shore sampling along the Victoria Land coast under the PNRA *aegida* (“XIX” expedition) took place by using a Rauschert dredge (Rehm et al. 2006, Ghiglione et al. 2013, Błażewicz-Paszkowycz and Siciński 2014). NIWA more recent expeditions (TAN0402 BIOROSS, 2004 and TAN0802 IPY-CAML, 2008) used VanVeen grab, epibenthic sled, Orange Roughy trawl, multicorer, and hyperbenthic sled. Samples from the “XXV” PNRA expedition originated form bottom samples collected by SCUBA divers by scraping the rock in cryptic environments such as crevices and holes present along the rocky cliff of Tethys Bay (“zecca” station). In more recent cruises (from 2003 on wards), all the collected specimens were fixed on board in at least 90% ethanol and brought back to the collections. After a general sorting, all the specimens were classified to the lowest taxonomical resolution by two expert taxonomists: Magdalena Błażewicz-Paszkowycz (Department of Polar Biology and Oceanobiology, University of Łódź, Poland) and Graham Bird (Independent, Kāpiti, New Zealand). The present tanaidaceans dataset has been formatted in order to fulfil the standards (Darwin Core) required by the OBIS scheme (http://iobis.org/data/schema-and-metadata) according to the SCAR-MarBIN Data Toolkit (available at http://www.scarmarbin.be/documents/SM-FATv1.zip). The dataset was uploaded in the ANTOBIS database (the geospatial component of SCAR-MarBIN).

All vouchers are now preserved in 75% ethanol and stored at the Italian National Antarctic Museum (MNA), Section of Genoa, Genoa (Italy) and at the National Institute of Water and Atmospheric Research (NIWA Invertebrate Collection), Wellington (New Zealand). Given the proper fixation of the material for molecular studies, a barcoding survey of the Tanaidacea from the Ross Sea is planned in the next future. The dataflow illustrating sampling, storing procedures and data/metadata availability is reported in Fig. 1.

**Quality control description:** Specimens were classified at the lowest possible taxonomic level and only those that have been classified at least at the genus level were included in the present dataset. During all the phases of sorting, classification and storage of samples, both at the Italian National Antarctic Museum and at the NIWA Invertebrate Collection, quality controls and data cleaning have been undertaken at various steps in order to produce quality data and make consistent cross-references between the database and samples’ labels (Fig. 1). Both MNA and NIWA use an SQL-based database (Specify 6) to manage their collections and link all the data (photos, sequences, etc.) to the physical samples. Georeferencing on board the R/V “*Italica*” is based on the interpolation of GPS satellite receivers (models 3S Navigation and Glonass ASHTECH GG24) and a gyrocompass. Station coordinates and sampling events were recorded during sampling activities through the “*Italica*” NetNav WEB system, which is based on the above GPS systems. On board the R/V “*Tangaroa*” a wide-area differential GPS system (models Seastar 9200 DGPS, Seastar 8200 DGPS) was used. Pre-GPS data have been reported as they appeared in original data reports (e.g. Bullivant and Dearborn 1967).

## Taxonomic coverage

**General taxonomic coverage description:** This dataset focused on the Order Tanaidacea (Kingdom Animalia, Phylum Arthropoda, Subphylum Crustacea, Superorder Peracarida) and include a total of 2953 specimens belonging to 33 genera and 50 different species. In the Southern Ocean, the order Tanaidacea numbers 160 species (Błażewicz-Paszkowycz 2013, De Broyer et al. 2014), thus representing the second most diverse group of benthic crustaceans after Isopoda and before Amphipoda (Appeltans et al. 2012, Błażewicz-Paszkowycz et al. 2012). In the Ross Sea, before Błażewicz-Paszkowycz and Siciński (2014), only eight tanaid species were known: seven of these reported by Sieg (1983, 1986) (*Nototanais dimorphus* (Beddard, 1886), *Andrognathia plumosa* Sieg, 1983, *Pseuodoparatanais antarcticus* Sieg, 1983, *Typhlotanaoides insolitus* Sieg, 1983, *Akanthophoreus antarcticus* (Vanhöffen, 1914), *Typhlotanais greenwichensis* Shiino, 1970, *Cryptocopoides antarctica* (Vanhöffen, 1914)) and one, *Exspina typica* (Vanhöffen, 1914) reported by Alvaro et al. (2011). It is worthy to mention that the taxonomical status of *Andrognathia plumosa* has been recently questioned, as the species is only known from a male and it might represent the male of another species, namely *Cryptocopoides antarctica* (see Błażewicz-Paszkowycz et al. 2014 for further details). Błażewicz-Paszkowycz and Siciński (2014), studying the materials collected with a Rauschert dredge (Rehm et al. 2006), reported 40 species for the area, of which only 5 had been previously recorded in the area, 14 represented new species and the remaining species were new records for the area.

### Taxonomic ranks

**Kingdom:** Animalia

**Phylum:** Arthropoda

**Subphylum:** Crustacea

**Class:** Malacostraca

**Superorder:** Peracarida

**Order:** Tanaidacea

**Genera:** *Akanthophoreus*, *Bathytanaissus*, *Chauliopleona*, *Collettea*, *Cryptocopoides*, *Exspina*, *Insociabilitanais*, *Leptognathia*, *Leptognathiella*, *Meromonakantha*, *Mimicarhaphura*, *Mirandotanais*, *Molotanaissus*, *Neotanais*, *Nototanais*, *Obesutanais*, *Parafilitanais*, *Paragathotanais*, *Paraleptognathia*, *Paranarthrura*, *Paratyphlotanais*, *Peraeospinosus*, *Protanaissus*, *Pseudoleptognathia*, *Pseudotanais*, *Pseudonototanais*, *Pseudoparatanais*, *Singula*, *Tanaella*, *Tanaopsis*, *Typhlotanais*, *Typhlotanaoides*, *Zeuxo*

**Species:** *Akanthophoreus antarcticus* (Vanhöffen, 1914); *Akanthophoreus australis* (Beddard, 1886); *Akanthophoreus multiserratus* (Hansen, 1913); *Akanthophoreus* sp.; *Bathytanaissus* sp.; *Chauliopleona nickeli* Guerrero-Kommritz, 2005; *Collettea antarctica* (Vanhöffen, 1914); *Cryptocopoides antarctica* (Vanhöffen, 1914), *Cryptocopoides* sp. A; *Exspina typica* Lang, 1968; *Insociabilitanais* sp.; *Leptognathia* sp.; Leptognathia aff. microcephala Kudinova-Pasternak, 1978; Leptognathia cf. breviremoides Sieg, 1986; *Leptognathia glandiceps* Shiino, 1978; Leptognathia cf. lineata Shiino, 1978; *Leptognathiella* sp.; Meromonakantha aff. macrocephala (Hansen, 1913); *Mimicarhaphura immanis* Sieg, 1986; *Mirandotanais vorax* Kussakin & Tzareva, 1974; *Molotanaissus makrotrichos* (Sieg, 1986); *Neotanais* sp.; *Nototanais antarcticus* (Hodgson, 1902); Nototanais cf. antarcticus (Hodgson, 1902); *Nototanais dimorphus* (Beddard, 1886); Nototanais cf. dimorphus (Beddard, 1886); *Obesutanais* sp. A; *Parafilitanais* sp. A; *Paragathotanais* sp. A; *Paraleptognathia multiserratoides* Guerrero-Kommritz, 2004; *Paranarthrura fortispina* Sieg, 1986; *Paratyphlotanais armatus* (Vanhöffen, 1914); *Peraeospinosus emergensis* Błażewicz-Paszkowycz, 2005; *Peraeospinosus subtigaleatus* Błażewicz-Paszkowycz, 2005; *Protanaissus longidactylus* (Shiino, 1970); *Pseudoleptognathia setosa* Sieg, 1986; Pseudonototanais (Pseudonototanais) bransfieldensis Sieg, 1986; *Pseudoparatanais brachycephalus* Sieg, 1986; *Pseudotanais* sp. A; *Singula* sp.; *Tanaella unisetosa* Sieg, 1986; *Tanaopsis kerguelenensis* Shiino, 1978; *Typhlotanais* sp.; *Typhlotanais* sp. B; *Typhlotanais* sp. C; Typhlotanais aff. mixtus Hansen, 1913; Typhlotanais aff. cornutus Sars, 1879; *Typhlotanais greenwichensis* Shiino, 1970; *Typhlotanaoides rostralis* (Tzareva, 1982); Zeuxo (Parazeuxo) phytalensis Sieg, 1980.

## Spatial coverage

### General spatial coverage

Ross Sea, Antarctica (Figure 2)

### Coordinates

PNRA V expedition: -74.748611S; 164.0875E

PNRA XI expedition: -74.7166 and -74.9065 S; 163.97467 and 164.12333E

PNRA XIV expedition: -74.78448S; 164.03895E

PNRA XIX expedition: -71.25833333 and -74.82166667S; 164.1916667 and 170.6983333E

PNRA XXV expedition: -74.69026667S; 164.10255E

NZOI expeditions: -77.5000 and -78.43330S; 166.16670 and 180.0000W

New Zealand TAN0402 BIOROSS voyage: -66.9136657 and -72.3153305S; 163.2246704 and 171.8278350E

New Zealand TAN0802 IPY-CAML voyage: -66.724000 and -76.775000S; 176.756000 and 178.828500E

### Temporal coverage

PNRA V expedition (1989/1990): January 5, 1990

PNRA XI expedition (1995/1996): January 31, 1996 – February 6, 1996

PNRA XIV expedition (1998/1999): February 4, 1999

PNRA XIX expedition (2003/2004): February 9, 2004 – February 21, 2004

PNRA XXV expedition (2009/2010): December 9, 2009 – December 26, 2009

NZOI expeditions (1958/1961): January 24, 1959 – January 31, 1961

New Zealand TAN0402 BIOROSS voyage (2004): February 04, 2004 – March 04, 2004

New Zealand TAN0802 IPY-CAML voyage (2008): February 11, 2008 – March 12, 2008

### Natural collections description

**Parent collection identifier:** Italian National Antarctic Museum (MNA Section of Genoa, Italy), and National Institute of Water and Atmospheric Research (NIWA Invertebrate Collection), Wellington (New Zealand)

**Collection name:** MNA (Section of Genoa) and NIWA Invertebrate Collection - Ross Sea Tanaidacea

**Collection identifier:** http://www.mna.it, http://niwa.co.nz/nic

**Specimen preservation method:** Recent material (i.e. those collected from 2003 on wards) was fixed in ethanol immediately after isolation, then sorted into morphospecies and placed in “Screw Thread Vials” (National Scientific, USA) or similar quality tubes and vials for further studies. Samples are now maintained in ethanol in the collections of the Italian National Antarctic Museum (MNA Section of Genoa, Italy) and at the National Institute of Water and Atmospheric Research (NIWA Invertebrate Collection, Wellington). Historical material (i.e. specimens collected before 2003) were generally fixed in formalin and then passed into ethanol for long-term storage. This latter group of species/specimens is therefore not usable for molecular analyses.

## Datasets

**Dataset description:** This dataset contains data about the Phylum Arthropoda, Subphylum Crustacea and Order Tanaidacea from the Ross Sea area. Combined, it includes 50 different species corresponding to a total of 2953 specimens. The validity and synonyms of each species name were checked in WoRMS (World Register of Marine Species; http://www.marinespecies.org; last accessed on 2014-07-23). The Darwin Core elements included in the dataset are: catalogue number (i.e. MNA and NIWA catalogue number), scientific name, station, latitude (DD), longitude (DD), date of collection (year, whenever possible), time of collection (day, whenever possible), event date, gear, institution code (i.e. the name of the institution where the samples are kept), collection code (i.e. MNA and NIWA acronyms), individual counts, basis of record and status. At present, the dataset does not include GenBankID codes referred to the samples, since sequencing will be done as a future step. Sequences will also be deposited in BOLDSYSTEMS (http://www.boldsystems.org/). Images at the electron microscope (SEM) will be made available through the ANTABIF “Antarctic Field Guide” project (http://afg.biodiversity.aq/) in the next months.

**Object name:** MNA (Section of Genoa) and NIWA Invertebrate Collection - Ross Sea Tanaidacea

**Character encoding:** UTF-8

**Format name:** Darwin Core Archive format

**Format version:** 6 (latest)

**Distribution:** http://ipt.biodiversity.aq/resource.do?r=mna_database_tanaidacea

**Language:** English

**Metadata language:** English

**License of use:** This dataset [MNA (Section of Genoa) and NIWA Invertebrate Collection - Ross Sea Tanaidacea] is made available under the Open Data Commons Attribution License: http://www.opendatacommons.org/license/by/1.0

**Date of metadata creation:** 2014-08-08

**Hierarchy level:** Dataset
